# The transcription factor TaMYB31 regulates the benzoxazinoid biosynthetic pathway in wheat

**DOI:** 10.1093/jxb/erac204

**Published:** 2022-05-12

**Authors:** Zhaniya S Batyrshina, Reut Shavit, Beery Yaakov, Samuel Bocobza, Vered Tzin

**Affiliations:** French Associates Institute for Agriculture and Biotechnology of Drylands, Jacob Blaustein Institutes for Desert Research, Ben-Gurion University of the Negev, Midreshet Ben Gurion, 8499000, Israel; French Associates Institute for Agriculture and Biotechnology of Drylands, Jacob Blaustein Institutes for Desert Research, Ben-Gurion University of the Negev, Midreshet Ben Gurion, 8499000, Israel; French Associates Institute for Agriculture and Biotechnology of Drylands, Jacob Blaustein Institutes for Desert Research, Ben-Gurion University of the Negev, Midreshet Ben Gurion, 8499000, Israel; Department of Ornamentals and Biotechnology, Institute of Plant Sciences, Agricultural Research Organization, The Volcani Center, 68 Hamakabim Road, 7528809, Rishon LeZion, Israel; French Associates Institute for Agriculture and Biotechnology of Drylands, Jacob Blaustein Institutes for Desert Research, Ben-Gurion University of the Negev, Midreshet Ben Gurion, 8499000, Israel; The James Hutton Institute, UK

**Keywords:** Abiotic stress, biotic stress, herbivores, *Rhopalosiphum padi* aphid, specialized metabolites, *Spodoptera littoralis* caterpillar, *Tetranychus urticae* spider mite, *Triticum aestivum*, Triticum turgidum

## Abstract

Benzoxazinoids are specialized metabolites that are highly abundant in staple crops, such as maize and wheat. Although their biosynthesis has been studied for several decades, the regulatory mechanisms of the benzoxazinoid pathway remain unknown. Here, we report that the wheat transcription factor MYB31 functions as a regulator of benzoxazinoid biosynthesis genes. A transcriptomic analysis of tetraploid wheat (*Triticum turgidum*) tissue revealed the up-regulation of two *TtMYB31* homoeologous genes upon aphid and caterpillar feeding. *TaMYB31* gene silencing in the hexaploid wheat *Triticum aestivum* significantly reduced benzoxazinoid metabolite levels and led to susceptibility to herbivores. Thus, aphid progeny production, caterpillar body weight gain, and spider mite oviposition significantly increased in *TaMYB31*-silenced plants. A comprehensive transcriptomic analysis of hexaploid wheat revealed that the *TaMYB31* gene is co-expressed with the target benzoxazinoid*-*encoded *Bx* genes under several biotic and environmental conditions. Therefore, we analyzed the effect of abiotic stresses on benzoxazinoid levels and discovered a strong accumulation of these compounds in the leaves. The results of a dual fluorescence assay indicated that TaMYB31 binds to the *Bx1* and *Bx4* gene promoters, thereby activating the transcription of genes involved in the benzoxazinoid pathway. Our finding is the first report of the transcriptional regulation mechanism of the benzoxazinoid pathway in wheat.

## Teaser

A combined transcriptomic, metabolic, and dual expression analysis was used to discover the MYB transcription factor that regulates benzoxazinoid biosynthesis under biotic and abiotic stresses in wheat.

## Introduction

In response to herbivore attacks, plants protect themselves with the production of a diverse array of specialized metabolites. These compounds function either as toxic deterrents to reduce insect feeding, repellents to limit herbivore visitations, or attractants to recruit natural enemies to locate their prey (Walling, 2000). The formation of specialized metabolites in response to stress is often regulated by the up-regulation of biosynthesis genes. Several transcription factors (TFs) are ­associated with the regulation of specialized metabolism, including members of the MYB, WRKY, and NAC families. MYB family members are involved in the biosynthesis of anthocyanin, phenylpropanoids, lignins, flavonoids, proanthocyanidins, phenolamides, and ethylene (Onkokesung *et al.*, 2012; Cao *et al.*, 2020; Ma *et al.*, 2021).

Benzoxazinoids (BXDs) are indole-derived specialized metabolites. They are abundant in monocot plant species from the grass family, such as wheat, maize, and rye (Frey *et al.*, 1997; Glawischnig *et al.*, 1999; Nomura *et al.*, 2002; Kokubo *et al.*, 2017), and in several dicot families (Frey *et al.*, 2009; Makowska *et al.*, 2015). Their biosynthesis pathway has mostly been investigated in maize (Makowska *et al.*, 2015; Niculaes *et al.*, 2018; Zhou *et al.*, 2018). Naturally occurring BXDs are divided into three groups based on their *N*-substituents: lactams, methyl derivatives, and hydroxamic acids (Cambier *et al.*, 2000). These metabolites represent the benzoxazinone class—basic metabolites of the biosynthesis pathway. Another BXD class is the benzoxazolinones—less toxic metabolites formed from benzoxazinones due to the loss of *N*-substituents (Wouters *et al.*, 2016). The first committed step toward BXD biosynthesis starts with indole-3-glycerolphosphate synthase (IGPS), which synthesizes indole-3-glycerol phosphate (Richter *et al.*, 2021). Indole-3-glycerol phosphate is then hydrolyzed by the indole-glycerol phosphate aldolase (Bx1), followed by four consecutive cytochrome P450 enzymes (Bx2–Bx5) and glucosyltransferases (Bx8/Bx9), resulting in the formation of DIBOA-Glc (2,4-dihydroxy-1,4-benzoxazin-3-one-glucoside). Further hydroxylation and subsequent methylation are catalyzed by Bx6–Bx7 and Bx10–Bx14 (Frey *et al.*, 1997; Rad *et al.*, 2001; Niemeyer, 2009; Meihls *et al.*, 2013; Handrick *et al.*, 2016). The glycosylated form of BXDs is stored in the vacuole and deglycosylated by β-glucosidases on demand (Niculaes *et al.*, 2018; Schütz *et al.*, 2019) (Supplementary Fig. S1).

The BXD compounds perform various functions. They have been intensively studied concerning their protection against biotic stresses, including herbivorous aphids and caterpillars (Niemeyer, 2009; Wouters *et al.*, 2016), mites (Bui *et al.*, 2018), fungal pathogens (Oikawa *et al.*, 2004; Søltoft *et al.*, 2008; Ahmad *et al.*, 2011), and root-associated microbiota (Hu *et al.*, 2018; Cotton *et al.*, 2019). BXDs also possess allelopathic properties in weed suppression (Quader *et al.*, 2001; Wu *et al.*, 2002). These compounds perform different roles beyond biotic stress protection, such as iron uptake (Tipton and Buell, 1970; Farkas *et al.*, 1998; Pethô, 2002), aluminum tolerance (Poschenrieder *et al.*, 2005), and possibly the regulation of flowering time (Romero Navarro *et al.*, 2017). Moreover, BXDs are involved in the indirect regulation of plant growth and development (Oikawa *et al.*, 2002; Kato-Noguchi, 2008) and stem elongation through interactions with plant hormones (Xu *et al.*, 2021).

BXD levels in plants are altered in response to environmental cues. For example, drought stress has been reported to increase the levels of 2,4-dihydroxy-7-methoxy-1,4-benzoxazin-3-one (DIMBOA) and 2,4-dihydroxy-1,4-benzoxazin-3-one (DIBOA) in maize leaves (Richardson and Bacon, 1993). In contrast, studies suggested that the *O*-methyltransferase, ZmBx12, which methylates DIMBOA-Glc into 2-hydroxy-4,7-dimethoxy-1,4-benzoxazin-3-one glucoside (HDMBOA-Glc), plays a role in the adaptation of maize plants to drought conditions (Zhang *et al.*, 2021b). A combination of drought and elevated atmospheric carbon dioxide has reduced HDMBOA-Glc levels, resulting in susceptibility to a mycotoxigenic pathogen (Vaughan *et al.*, 2016). In wheat seedlings, the BXD levels were only affected by temperature changes (15, 20, and 25 °C), which were suggested to be at least partially mediated through their effect on plant growth (Gianoli and Niemeyer, 1996, 1997). Rye plants have shown a reduction in BXD biosynthesis under prolonged low-temperature conditions in biochemical and gene expression assays (Stochmal and Kowalczyk, 2020). Taken together, this suggests that BXDs participate in biotic and abiotic stress responses, as well as developmental processes.

Although BXD metabolism has been studied for several decades, the transcriptional regulation of *Bx* genes has not yet been thoroughly investigated. Only a handful of reports indicate the potential TFs that might be involved in regulating this pathway in maize plants. The Mo17 and B73 inbred maize lines showed induction of the gene expression levels of four TF genes (*ZmWRKY75*, *ZmMYB61*, *ZmNAC35*, and *ZmGRAS37*) upon *Rhopalosiphum padi* aphid infestation, which was implicated in the up-regulation of the *ZmBx1* and *ZmBx13* genes and positively correlated with BXD abundance (Song *et al.*, 2017). Another report suggested that *ZmbHLH57* and *ZmWRKY34* may regulate *Bx* genes in systemic leaves stimulated by the *Mythimna separata* oriental armyworm (Malook *et al.*, 2019). An analysis of multiple gene regulatory networks in maize identified four TFs that have been suggested to regulate *Bx* genes, including TF families such as MYB (*ZmMYB112*), NAC (*ZmNAC21*), GRAS (*dwarf plant 8*), and G2-like (*golden plant 2*) (Zhou *et al.*, 2020). However, these studies were performed using maize transcriptomic approaches without directly testing the function of the identified TFs. Thus far, only ZmbHLH20 and ZmbHLH76 (basic helix–loop–helix) were functionally determined as regulators of BXD biosynthesis using a protoplast transfection system (Gao *et al.*, 2019). However, the function of TFs and their interaction with *Bx* genes *in planta* remain unexplored.

In this study, we investigated the transcriptional regulation of BXD biosynthesis-related genes in wheat. Our recent transcriptomic analysis of the tetraploid wheat (*Triticum turgidum* ssp. *durum*) cv. Svevo wheat transcriptome revealed the up-regulation of *Bx* genes upon 6 h of *Spodoptera littoralis* caterpillar feeding (Shavit *et al.*, 2022). We exploited this transcriptomic dataset to determine which TFs are overexpressed in response to herbivore attacks. Then, we evaluated the possible role played by the TaMYB31 TF using a *Barley stripe mosaic virus* (BSMV)-based virus-induced gene silencing (VIGS) system and found that TaMYB31 is involved in the regulation of BXDs in wheat seedlings. Furthermore, *TaMYB31*-silenced wheat plants were more susceptible to herbivorous insects than the control plants. Following a previous study that reported that the *TaMYB31* gene is involved in drought responses in wheat (Bi *et al.*, 2016), we hypothesized that BXD levels would also be induced under drought conditions. Thus, we performed several abiotic stress experiments, including drought, polyethylene glycol (PEG), cold, and salt stresses, and discovered that BXD levels increased under these conditions. Lastly, we investigated the *Bx* binding selectivity of TaMYB31 by ectopic expression in BXD-deficient tobacco plants and identified the promoters of several *Bx* genes activated by this TF. This is the first report to suggest that TaMYB31 regulates the biosynthesis of BXDs and plays a role under both biotic and abiotic stresses.

## Materials and methods

### Plant material and growth conditions

Hexaploid wheat (*Triticum aestivum* L. cultivar Chinese Spring) and tobacco (*Nicotiana benthamiana*) were used in this study. Seeds were germinated in plastic pots (330 cm^3^) containing a moistened tuff mixture with vermiculite (2:1) and an N–P–K fertilizer (20–20–20); they were then kept in a growth room and maintained under a controlled regime of 24–26 °C and 16/8 h of light/dark, and were watered as needed.

### Extraction of DNA and RNA

The second leaf of 11-day-old wheat plants was harvested, flash-frozen in liquid nitrogen, and stored at –80 °C. The frozen leaf tissue was ground into a fine powder using a Retsch Mixer Mill MM 400 (ProfiLab24 GmbH, Germany) with pre-chilled holders and grinding beads, and was used for different analyses. A Plant Genomic DNA Mini Kit (Geneaid Biotech Ltd, Taiwan) was used for genomic DNA isolation. RNA was extracted using a Spectrum™ Plant Total RNA Kit (Sigma-Aldrich, USA), according to the manufacturer’s instructions, and treated with Dnase I (Thermo Fisher Scientific. Inc., USA). For quantitative reverse transcription–PCR (qRT–PCR), purified RNA was quantified and checked for integrity by electrophoresis on a 2% agarose gel and by a NanoDrop One-W UV-Vis spectrophotometer (Thermo Fisher Scientific, USA). Then, 1 µg of RNA, extracted from the wheat leaves, was used to synthesize cDNA using the qScript cDNA Synthesis Kit (Quantabio, USA).

### Virus-induced gene silencing cloning and assay

The cDNA from the hexaploid wheat Chinese Spring cultivar was used to amplify the coding sequence fragment of the *TaMYB31* gene (GenBank accession KU674897; IWGSC gene IDs: TraesCS5A02G227400, TraesCS5B02G226100, and TraesCS5D02G234800). A fragment of 213 bp was cloned into the BSMV RNAi vector pCa-ybLIC via a ligation-independent cloning method, using *Apa*I restriction enzyme (Yuan *et al.*, 2011; Lee *et al.*, 2015), and transformed into *Agrobacterium tumefaciens* strain GV3101. A fragment of the phytoene desaturase (*PDS*) gene was fused into the BSMV system and used as a positive control showing the photobleaching phenotype (Travella *et al.*, 2006; Yuan *et al.*, 2011; Feng *et al.*, 2015; Lee *et al.*, 2015). The final optical density of agrobacteria, carrying target genes, was brought to OD_600_=1.0 in an infiltration buffer containing 10 mM MgCl_2_, 10 mM MES at pH 5.6, and 150 µM acetosyringone. Resuspended cells were incubated at room temperature for 3 h and mixed in a 1:1:1 ratio with BSMV RNAα- and RNAβ-carrying agrobacteria before infiltration, and then pressure-infiltrated into *N. benthamiana* leaves. Five days post-agroinfiltration, virus-infected *N. benthamiana* leaves were homogenized (1:3 w:v) with 10 mM potassium phosphate buffer (pH 7) containing 1–2% w/v Celite^®^ 545 AW and the sap was inoculated into 5-day-old first wheat leaves. As a negative control of gene silencing, an empty BSMV vector (without any gene insert) was used. Finally, 14 days post-inoculation (dpi), the second leaves were harvested and used for several analyses, including metabolic measurements, gene expression analysis, and insect performance assays. The list of primers used for the cloning is presented in Supplementary Table S1.

### Quantitative RT–PCR analysis

For the amplification of the *TaMYB31* gene fragment, primers were designed using the Primer3Plus software (www.bioinformatics.nl/cgi-bin/primer3plus/primer3plus.cgi/). The specificity of the primers was confirmed by agarose gel electrophoresis and melting curve analysis. The efficiency of the primers (90–110%) was determined by a standard curve with a 3-fold serial dilution of cDNA. Two *T. aestivum* genes, *Glyceraldehyde-3-phosphate dehydrogenase* (*GAPC*) and *Actin* (*ACT-1*), were used as reference genes. Both genes were previously used for the normalization of expression of *TaMYB31* in wheat and Arabidopsis plants under drought conditions (Bi *et al.*, 2016; Zhao *et al.*, 2018) and for quantifying *BSMV* gene silencing efficiency in wheat (Zhai *et al.*, 2017; Shavit *et al.*, 2022). The cDNA of VIGS-inoculated plants was prepared as described above. Reactions were prepared in triplicate for each sample with Power SYBR^®^ Green PCR Master Mix (Applied Biosystems™, USA) and run on a 7500 Real-Time PCR System (Applied Biosystems™, USA). The PCR was initiated by incubation at 95 °C for 10 min, and amplification was performed in 40 cycles (95 °C for 30 s and 60 °C for 15 s). For each gene, four biological replicates were analyzed for *TaMYB31*-silenced plants and compared with the *BSMV::empty* vector. The list of primers used for the qRT–PCR is shown in Supplementary Table S1.

### Network analysis

For the enrichment analysis of *MYB31*/*Bx* genes in wheat, *Bx* genes were manually curated from various studies (Nomura *et al.*, 2005; Sue *et al.*, 2011, 2021; Li *et al.*, 2018; Richter *et al.*, 2021) and by BLAST sequence alignment against annotated maize genes (Altschul *et al.*, 1990). Next, a 1 kb segment of the genomic DNA sequence upstream of the transcription start site of each gene was extracted, and a prediction of TF binding was performed using the PlantRegMap binding site prediction tool (planttfdb.cbi.pku.edu.cn) against *T. aestivum* (threshold *P*-value ≤10^−4^). The *Bx* genes were selected by their predicted binding of TaMYB31 for further analysis (Supplementary Table S2). Next, wheat expression datasets (TPM values) were collected from the Earlham Institutes Grassroots data repository for *T. aestivum* genes RefSeq v1.1 (opendata.earlham.ac.uk/wheat); (Ramírez-González *et al.*, 2018). Then, 18 out of the 35 datasets relevant to the current experimental design were selected. We prioritized expression data from wheat above-ground tissue, early stages of development, diseases such as pathogenic fungi and powdery mildew, and the following abiotic stress conditions: phosphorus starvation, drought, heat, and cold (Supplementary Table S3). Results from the scale-free topology (soft threshold) and subsequent weighted gene co-expression network analysis (WGCNA) analysis for the 14 datasets were examined (Supplementary Table S4), including the total number of modules in the dataset (modules), the total number of genes in the dataset (total genes), the total number of unassigned genes (grey module genes), and the number of unassigned *Bx* genes (grey module *Bx* genes). These datasets were clustered using the WGCNA R package (v1.70-3) (Zhang and Horvath, 2005; Langfelder and Horvath, 2008), after which three sets were discarded due to unreliable results in the pickSoftThreshold function, and one set failed the TOMsimilarity/adjacency functions due to memory limitations. Of the remaining 14, only six sets had modules with enriched *MYB31*/*Bx* genes. Enrichment analysis of *MYB31*/*Bx* genes in each dataset was conducted by a Fisher’s exact test (Fisher, 1970; Benjamini and Yekutieli, 2001) in R (v4.1.0; R Core Team, 2021)

### Benzoxazinoid extraction and analysis

BXDs were extracted from the upper part of the second leaves of VIGS-inoculated plants. Samples were extracted as previously described (Shavit *et al.*, 2018). In brief, tissues were ground to a fine powder and mixed with an extraction solvent of 80% methanol and 0.1% formic acid in double-distilled water (DDW) in a 1:10 (w:v) ratio. Benzoxazolin-2(3H)-one (BOA; Sigma-Aldrich, USA) was added to the extraction buffer as an internal standard to a final concentration of 10 µg ml^–1^. Then, samples were briefly vortexed, sonicated in ice for 40 min, filtered with a 0.22 µm sterilizing membrane (EMD Millipore Corp., USA), and kept at 4 °C before analysis. Metabolite separation was conducted on a Dionex UltiMate 3000 HUPLC system using a C18 reverse-phase Hypersil GOLD column with 3 μm pore size, 150 × 4.60 mm (Thermo Fisher Scientific, Germany). The sample-running protocol followed instrumental conditions described previously (Batyrshina *et al.*, 2020) with 5 µl per sample injection. Quantification was done on Chromeleon software (Thermo Fisher Scientific Inc.) by confirming metabolites using UV spectra and standards (Shavit *et al.*, 2022).

### Abiotic stress conditions

For stimulating drought stress, Chinese Spring wheat seeds were sown on moistened soil and grown in 330 cm^3^ pots in growth room conditions. Plants were watered after 14 d, and tissues were harvested 2 d after the second watering. For the dehydration stress, sterilized seeds were grown on PEG-infused plates (Verslues *et al.*, 2006) with –1.2 MPa final water potential. A half-strength Murashige and Skoog (MS) medium was used to grow control plants (Murashige and Skoog, 1962). The seed sterilization procedure included washing seeds in a 3% sodium hypochlorite solution for 10 min, followed by rinsing three times with sterile water. To stimulate salt and low-temperature stresses, plants were grown in Petri dishes and shared the same control grown on DDW. Salt-treated plants were grown in a 150 mM NaCl solution, while low-temperature testing plates received DDW. For low-temperature treatments, 5-day-old seedlings were grown in favorable growth conditions and then transferred to 4 °C in a cold room for 2 d. Shoot tissues were harvested from each plant, with at least seven biological replicates per treatment, and kept at –80 °C prior to metabolic analyses.

### Aphid, caterpillar, and mite bioassays

Pests were maintained in controlled growth room conditions under a 16 h light and 8 h dark photoperiod at 24–26 °C. All insect bioassays were tested on second leaves (only the first leaf was infected with VIGS plasmids) and applied on VIGS-inoculated plants at 10 dpi. Insect bioassays were performed on a *BSMV::empty* vector as a control treatment. The bird cherry-oat aphid (*R. padi*) colony was reared on 2- to 3-week-old bread wheat plants (*T. aestivum* cv. Rotem; Agridera Seeds & Agriculture Ltd, Israel). The aphid bioassay was conducted by confining 10 adult *R. padi* aphids inside a clip cage (4.5 cm in diameter) attached to the upper part of the second leaf. Aphid progeny production was counted after 96 h of infestation. The Egyptian cotton leafworm (*S. littoralis*) second and third instars were provided by Dr Rami Horowitz (ARO, Israel) and were fed for 2 d on 2-week-old Chinese Spring seedlings prior to the experiment. For the caterpillar bioassay, the second leaf of 15-day-old Chinese Spring plants (VIGS-inoculated plants at 10 dpi) was covered with a breathable cellophane bag in which *S. littoralis* larvae were applied. After 3 d, the caterpillar’s body weight was measured. Two-spotted spider mite (TSSM; *Tetranychus urticae*) eggs were obtained from Biobee Sde Eliyahu Ltd (Israel) and maintained on 4- to 5-week-old wheat plants (*T. aestivum* cv. Rotem). To remove the possible maternal effects of host plants, mites were acclimated to wheat plants at least three generations before the experiment. For the TSSM oviposition assay, a female was placed on the abaxial side of a wheat leaf segment (4 cm long) and kept on water-saturated cotton wool in a plastic container under growth room conditions, and eggs were counted after 3 d, under a binocular microscope (Weinblum *et al.*, 2021). In total, 12 leaf segments were tested per treatment.

### Dual expression system using transient expression in tobacco leaves

To test the candidate promoters, an ~1 kb sequence upstream of the start codon of the selected *Bx* genes was amplified by PCR from *T. aestivum* Chinese Spring genomic DNA, using Phusion High-Fidelity DNA Polymerase (PCRBIO Verifi™ Polymerase, PCR Biosystems Ltd, UK). The coding region of the *TaMYB31* gene was fully synthesized by Genewiz (Gene Synthesis Services, South Plainfield, NJ, USA) using a codon-optimized sequence to eliminate the inner IIS restriction recognition sites (*Bsa*I and *Bsm*BI; Supplementary Fig. S2). To assess whether TaMYB31 enhances the expression of *Bx*-associated genes *in planta*, a dual expression system based on transient gene expression in tobacco (*N. benthamiana*) leaves was established. The Goldenbraid (GB) cloning system was used for contract building, and cassettes were built using a multipartite assembly (Sarrion-Perdigones *et al.*, 2013). Plasmids were generated to include the putative promoter sequences of the *Bx* genes, combined with green fluorescent protein (GFP) as a reporter module, *TaMYB31* driven by the *Cauliflower mosaic virus* 35S promoter, and the red fluorescent protein (RFP) driven by the Arabidopsis *Ubiquitin10* (*AtUBQ10*) promoter, which was used as an internal standard for normalization of the results. A plasmid used as a negative control to measure background promoter activity was similarly generated, except for the *TaMYB31* gene, which was not included. As a positive control, *TaKCS1* (3-ketoacyl CoA synthetase promoter, KU737579), which was previously reported to be a target gene for TaMYB31 under drought conditions (Bi *et al.*, 2016), was also tested. The final vectors were transferred to *A. tumefaciens* strain GV3101 using the freeze–thaw transformation method (Hofgen and Willmitzer, 1988). For tobacco agroinfiltration, an overnight-grown culture was resuspended in infiltration buffer, as described previously in the VIGS assay, and incubated for 3 h. After inoculation, plants were maintained in a growth room until infiltrated leaves were collected at 4 dpi. The list of primers used for the cloning is given in Supplementary Table S1.

### Protein extraction and fluorescence assay

Tobacco leaf tissues were harvested and ground with a mortar and pestle in liquid nitrogen. Then, 100 mg of homogenized tissue powder was mixed with an extraction buffer containing 10 mM TES pH 7.4, 300 mM sucrose, and 1× protease inhibitor in a 1:4 ratio, and centrifuged for 10 min at 4 °C at 14 000 *g*. The supernatant was collected and centrifuged again for 25 min at 4 °C at 14 000 *g*. The recovered supernatant was used for the Bradford protein assay (Bradford, 1976). Accordingly, 1 μg of crude protein extract from each sample was used for fluorescent protein analysis, and fluorescence measurements were performed in the Tecan Infinite M200 microplate reader (Tecan, Switzerland). The test was set with the following parameters: for GFP intensity, the excitation wavelength was 488 nm, and the emission wavelength was 525 nm; and for RFP intensity, the excitation wavelength was 590 nm, and the emission wavelength was 635 nm, respectively. The assay was repeated three times with five biological replicates per construct.

### Statistics

The Student’s *t*-test was used for paired comparisons of treatments with controls. All tests were performed in JMP software (SAS) and figures were designed using Microsoft Excel.

## Results

### Selecting candidate transcription factors modified in response to insect feeding

To understand the regulatory mechanism underlying BXD biosynthesis, we exploited the recently published transcriptome dataset of tetraploid wheat, where several *Bx* genes were up-regulated under insect attack (Shavit *et al.*, 2022). This dataset identified TF candidates from the nine TF families (MYB, NAC, WRKY, bHLH, GRAS, bZIP, GLABROUS1, BTB/POZ, and heat shock factors), potentially regulating specialized metabolism. We identified 17 TFs that were up-regulated upon caterpillar feeding. *Bzip9*, *TaMYB31*, *TaMYB29*, *TaNAC41*, *TaWRKY36*, BTB/POZ/TAZ domain-containing protein-2, and HSF clone *HD2967* were also induced upon aphid infestation, although the last two transcripts did not show significant changes. As shown in Table 1, mainly the subgenome B homeologs of the TF genes were induced under insect feeding.

**Table 1.** Gene expression of transcription factors induced by herbivory

*n*	Transcription factor name	Svevo v1. Ensemble ID	IWGSC Ensemble ID	Aphids/control		Caterpillars/control	
				log2FC	*P*-value	log2FC	*P*-value
1	bHLH	TRITD1Av1G205210	TraesCS1A02G362800	0.69	3.6E-01	7.26	**9.1E-27**
		TRITD1Bv1G195110	TraesCS1B02G380000	–2.13	1.5E-01	4.77	**1.1E-07**
			TraesCS1D02G367700				
2	BTB/POZ and TAZ domain-containing protein 2	TRITD2Av1G206590	TraesCS2A02G323200	0.90	6.0E-01	8.42	**3.1E-11**
		TRITD2Bv1G170350	TraesCS2B02G359400	1.28	2.6E-01	4.07	**1.3E-04**
3	bZIP (OBF1c)	TRITD6Av1G059270	TraesCS6A02G154600	0.18	5.9E-01	0.21	5.3E-01
		TRITD6Bv1G068030	TraesCS6B02G182500	0.30	4.8E-01	1.79	**2.3E-05**
			TraesCS6D02G144400				
4	bZIP9	TRITD7Av1G054450	TraesCS7A02G170600	0.89	**4.2E-02**	1.45	**8.3E-04**
		TRITD7Bv1G030080	TraesCS7B02G075600	–0.36	6.5E-01	1.89	**1.3E-02**
			TraesCS7D02G171300				
5	GRAS putative	TRITD4Av1G155270	TraesCS4A02G191300	0.07	8.5E-01	1.02	**6.5E-03**
		TRITD4Bv1G051570	TraesCS4B02G124000	0.57	2.2E-01	0.65	1.6E-01
			TraesCS4D02G122000				
6	Hsf clone HD2967	TRITD7Av1G195490	TraesCS7A02G360400	–0.07	9.1E-01	2.75	**4.5E-05**
		TRITD7Bv1G150310	TraesCS7B02G267300	1.26	2.1E-01	5.30	**5.2E-08**
7	HsfA4e	TRITD2Av1G076140	TraesCS2A02G204500	–0.05	9.4E-01	3.20	**6.9E-08**
		TRITD2Bv1G084940	TraesCS2B02G232000	–0.45	6.5E-01	5.27	**6.0E-10**
			TraesCS2D02G211400				
8	MYB31	TRITD5Av1G144700	TraesCS5A02G227400	–0.11	8.6E-01	1.51	**1.2E-02**
		TRITD5Bv1G134610	TraesCS5B02G226100	1.09	**1.3E-02**	1.37	**1.6E-03**
			TraesCS5D02G234800				
9	NAC41	TRITD5Av1G170450	TraesCS5A02G291200	1.09	**5.7E-05**	1.61	**1.8E-09**
		TRITD5Bv1G161590	TraesCS5B02G290200	1.11	**5.0E-03**	-0.24	5.5E-01
			TraesCS5D02G298600				
10	STOREKEEPER/GeBP		TraesCS7A02G199000				
		TRITD7Bv1G043720	TraesCS7B02G105700	–0.06	9.1E-01	1.55	**4.2E-03**
			TraesCS7D02G201700				
11	WRKY clone8	TRITD1Av1G209580	TraesCS1A02G401800	–0.99	2.7E-01	2.42	**1.6E-03**
		TRITD1Bv1G214620		–0.90	4.1E-01	2.51	**6.8E-03**
12	WRKY27	TRITD2Av1G202930	TraesCS2A02G330500			4.83	**8.3E-04**
			TraesCS2B02G351600				
			TraesCS2D02G332300				
13	WRKY28	TRITD3Av1G184990	TraesCS3A02G281900			5.03	**3.3E-04**
		TRITD3Bv1G167040	TraesCS3B02G315600	0.43	6.3E-01	2.74	**1.2E-03**
			TraesCS3D02G281900				
14	WRKY36	TRITD3Av1G150670	TraesCS3A02G228600	–1.14	3.4E-01	4.58	**1.0E-07**
		TRITD3Bv1G133450	TraesCS3B02G256000	2.84	**8.4E-04**	4.66	**2.0E-08**
			TraesCS3D02G226300				
15	WRKY64	TRITD1Av1G222800	TraesCS1A02G421900	0.35	5.5E-01	4.35	**2.9E-17**
			TraesCS1B02G453200				
			TraesCS1D02G429800				
16	WRKY68	TRITD2Av1G251140	TraesCS2A02G433000	–0.41	2.1E-01	1.19	**3.6E-04**
		TRITD2Bv1G212960	TraesCS2B02G454300	0.24	5.4E-01	1.00	**1.0E-02**
			TraesCS2D02G431000				
17	MYB29	TRITD5Av1G185470	TraesCS5A02G329900	–0.36	4.3E-01	–0.06	8.9E-01
		TRITD5Bv1G175020	TraesCS5B02G330100	1.94	**2.1E-03**	1.97	**1.8E-03**
			TraesCS5D02G335700				

Wheat seedlings (tetraploid durum wheat Svevo cultivar) were infested with *R. padi* and *S. littoralis* for 6 h. Shown are fold change values relative to the uninfested control in log2. *P*-values in bold represent *P*<0.05. The full description of the RNA-seq data and the experimental setup are presented in Shavit *et al.* (2022)

To analyze the interaction between TFs and *Bx* promoters, we performed *in silico* prediction using PlantRegMap. The following criteria were applied to select TF candidates: (i) TF family function analysis (Supplementary Table S5) and (ii) *in silico* prediction of interaction with *Bx* promoters. Most candidate TFs (Supplementary Table S6) did not show a possible interaction with *Bx* promoters, apart from TaMYB31, TaWRKY68, bZIP9, and TaMYB29 (Supplementary Table S7). Thus, with at least one binding site on each promoter, TaMYB31, TaMYB29, and TaWRKY68 were predicted to interact with the promoters of 25, 17, and 16 *Bx* genes, respectively, whereas the prediction for bZIP9 resulted in four homoeologous promoters located upstream of *Bx5* and *Bx6*. In this study, we focus on TaMYB31 for further functional analysis using a transient silencing system.

### Silencing the transcription factor gene *TaMYB31* affected benzoxazinoid biosynthesis in wheat

**Fig. 1.**
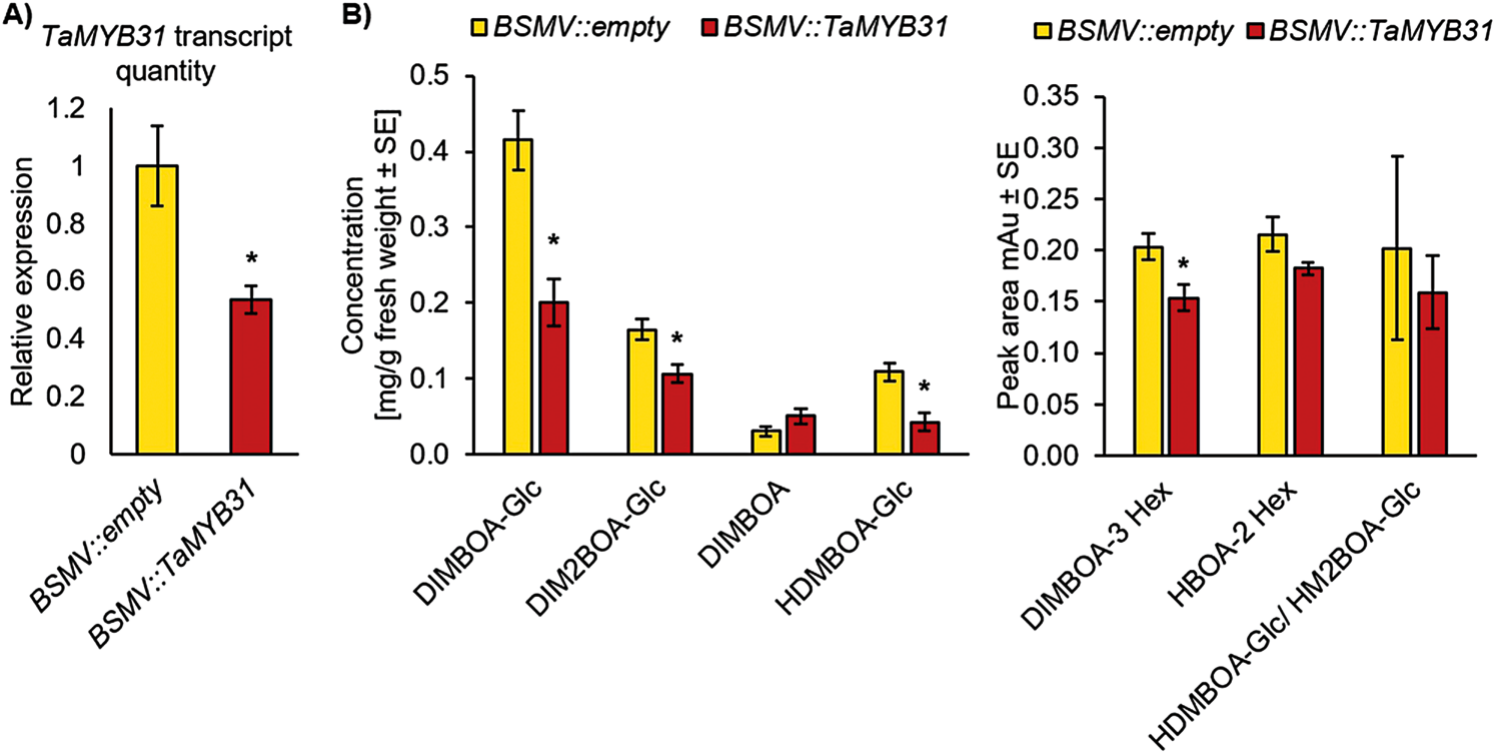
Effects of *TaMYB31* gene silencing on benzoxazinoid content. RNA and metabolites were extracted from the second leaves of wheat plants at 14 dpi BSMV-VIGS containing the *TaMYB31* gene. (A) Expression of *TaMYB31* by qRT–PCR using two reference genes *Glyceraldehyde-3-phosphate dehydrogenase* (*GAPC*) and Actin (*ACT-1*) (*n*=4). (B) Left panel, BXD metabolite levels presented as mg g^–1^ FW. Right panel, BXD metabolite levels in peak area from chromatogram (*n*=10–11). The comparison was made using Student’s *t*-test relative to *BSMV::empty* vector, *P*<0.05. Asterisks indicate a significant difference. In this figure, a single independent biological experiment is presented.

To investigate whether the selected TF gene *TaMYB31* is involved in BXD biosynthesis, we used the BSMV-based VIGS system (Lee *et al.*, 2015). The effect of *TaMYB31* gene silencing was confirmed by measuring the BXD levels in leaves. As a positive control of the VIGS system, a few plants were infected with *BSMV::PDS*, and a leaf photobleaching phenotype was detected (Supplementary Fig. S3). The silencing efficiency of the *TaMYB31* gene was verified by qRT–PCR analysis relative to two reference genes *GAPC* and *ACT-1*. The results showed that the expression level of *TaMYB31* was reduced by 46% in silenced plants compared with the *BSMV::empty* vector (Fig. 1A). As a result of this gene silencing, the levels of several BXD compounds, including DIMBOA-Glc, DIM2BOA-Glc, HDMBOA-Glc, and DIMBOA-3 Hex, were significantly decreased relative to the empty vector control (Fig. 1B). This metabolic analysis suggests that *TaMYB31* is a potential regulator of the BXD pathway.

### *TaMYB31* is involved in defense against insect herbivory

**Fig. 2.**
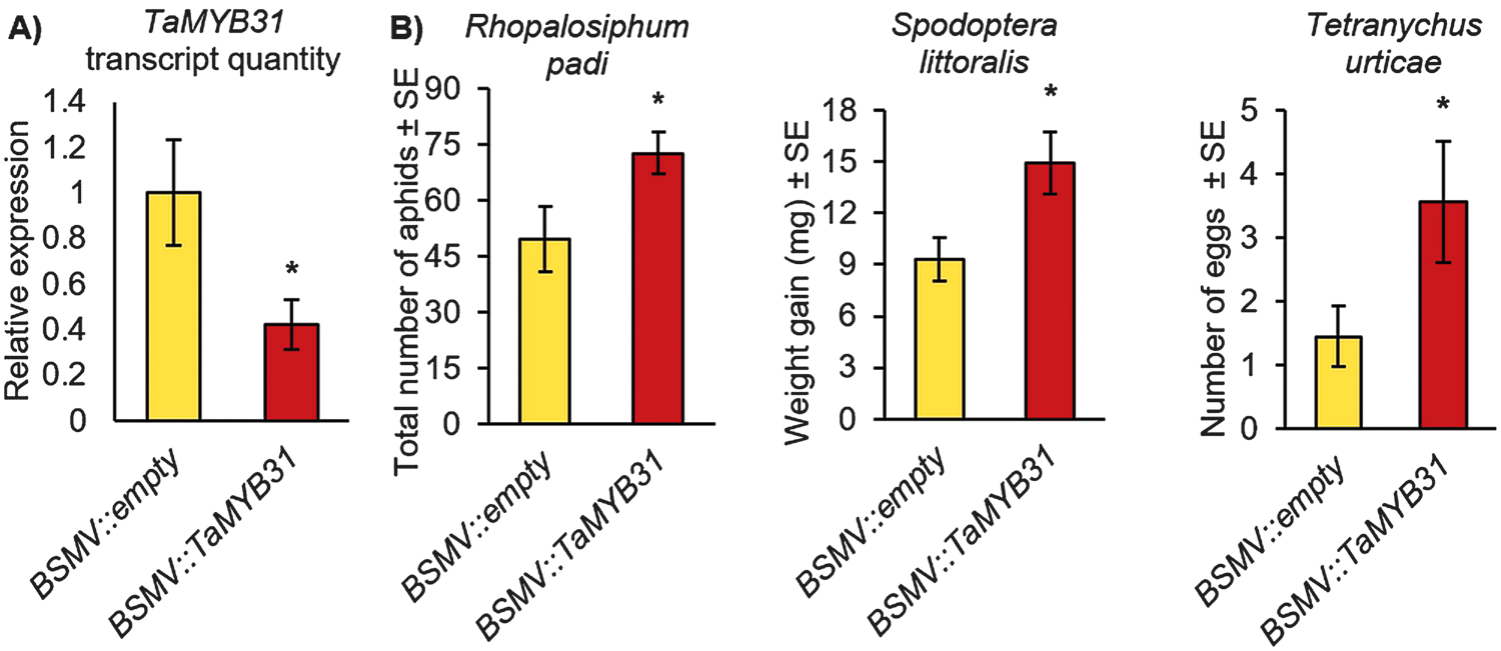
Gene silencing of *TaMYB31* affects insect herbivory susceptibility. Insect bioassay was performed on BSMV-VIGS-inoculated plants. (A) Expression of *TaMYB31* by qRT–PCR using two reference genes *Glyceraldehyde-3-phosphate dehydrogenase* (*GAPC*) and Actin (*ACT-1*) (*n*=5). Data are presented as means from a single independent biological experiment ±SE. Asterisks indicate a significant difference, Student’s *t*-test *P*<0.05. (B) Aphid (*Rhopalosiphum padi*) progeny production after 4 d (*n*=5); caterpillar (*Spodoptera littoralis*) weight after 3 d (*n*=10); two-spotted spider mite (*Tetranychus urticae*) oviposition after 3 d (*n*=12).

To evaluate whether *TaMYB31* expression plays a role in biotic stresses, several bioassays of pest infestation from different feeding guilds were conducted, including (i) a phloem feeder, the bird cherry-oat aphid (*R. padi*), (ii) a leaf-chewing insect, the Egyptian cotton leafworm caterpillar (*S. littoralis*), and (iii) a cell content feeder, the two-spotted spider mite (TSSM; *T. urticae*). As shown in Fig. 2A, the expression level of *TaMYB31* in the *BSMV::TaMYB31* plants was reduced by 57%. As a result, these plants were more susceptible to all three types of pests than the *BSMV::empty* control plants (Fig. 2B). This emphasizes the vital role this TF plays in wheat defense mechanisms, which may result from BXD reduction. Thus, we measured the BXD levels of *TaMYB31*-silenced plants after herbivore feeding and compared them with the *BSMV::empty* vector. As shown in Supplementary Fig. S4, aphid feeding did not affect the BXD levels of either *BSMV::empty* or *BSMV::TaMYB31* plants, while significant changes were detected upon caterpillar attack. The BXD compounds, DIMBOA-3 Hex, HBOA-2 Hex, and DIMBOA-Glc/DIMBOA were dramatically reduced in *TaMYB31*-silenced plants after caterpillar feeding. In contrast, only the reduction of DIMBOA-Glc/DIMBOA was observed in *BSMV::empty* plants, while HDMBOA-Glc was significantly increased. These results support previous studies that showed similar trends of BXD metabolites upon caterpillar feeding in maize and wheat plants (Tzin *et al.*, 2015; Li *et al.*, 2018).

### TaMYB31 protein activates *Bx* gene promoters

**Fig. 3.**
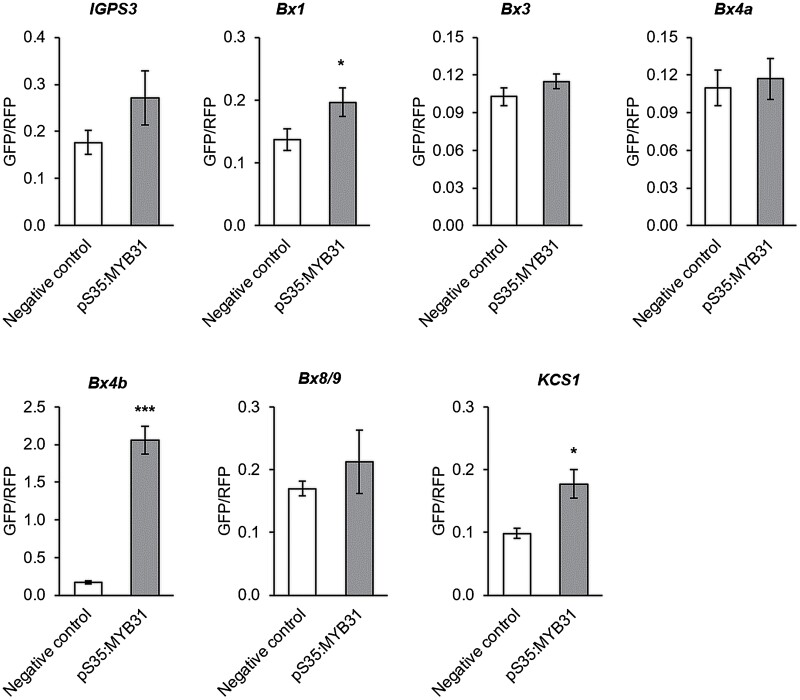
*TaMYB31* activates promoters of genes from the benzoxazinoid pathway. *TaMYB31* protein and *Bx* gene promoter interactions were evaluated using a dual fluorescence system based on transient expression. Background promoter activity was assayed by infiltration of the same empty vector for each gene without a *TaMYB31* cassette. Promoter sequences of the following genes were analyzed: *IGPS3* (TraesCS7B02G423900), *Bx1* (TraesCS7B02G294800), *Bx3* (TraesCS5B02G007200), *Bx4a* (TraesCS5A02G008800), *Bx4b* (TraesCS5B02G007100), *Bx8/9* (TraesCS7B02G016800), and *TaKCS1* (KU737579). Shown are means of the GFP/RFP reporters ratio ±SE. Asterisks indicate a significant difference by Student’s *t*-test: *P<0.05; ***P<0.001 (*n*=5). In this figure, a single independent biological experiment is presented.

To evaluate the physical interaction of TaMYB31 and *Bx* gene promoters, we took advantage of a dual fluorescence expression system using transient expression in tobacco leaves. Six selected promoters of *Bx*-related genes were cloned and fused to the *GFP* reporter gene and assembled with the *pro-AtUBQ10::RFP* cassette. The resulting vectors were used as negative controls to determine the activity of promoters in the absence of an exogenous activator. Additionally, these cassettes were also fused to the *pS35::TaMYB31* cassette into a final vector, and the GFP/RFP ratio was measured. As presented in Fig. 3, TaMYB31 activated the promoters of *Bx1* ­(TraesCS7B02G294800) and *Bx4b* (TraesCS5B02G007100). No changes in fluorescence were obtained using the ­promoters of *Bx3* (TraesCS5B02G007200), *Bx4a* ­(TraesCS5A02G008800), *Bx8/9* (TraesCS7B02G016800), or *IGPS3* (TraesCS7B02G423900). Overall, these results suggested that the TaMYB31 TF may activate some of the *Bx* promoters.

### Benzoxazinoid biosynthesis is induced under abiotic stress conditions

**Fig. 4.**
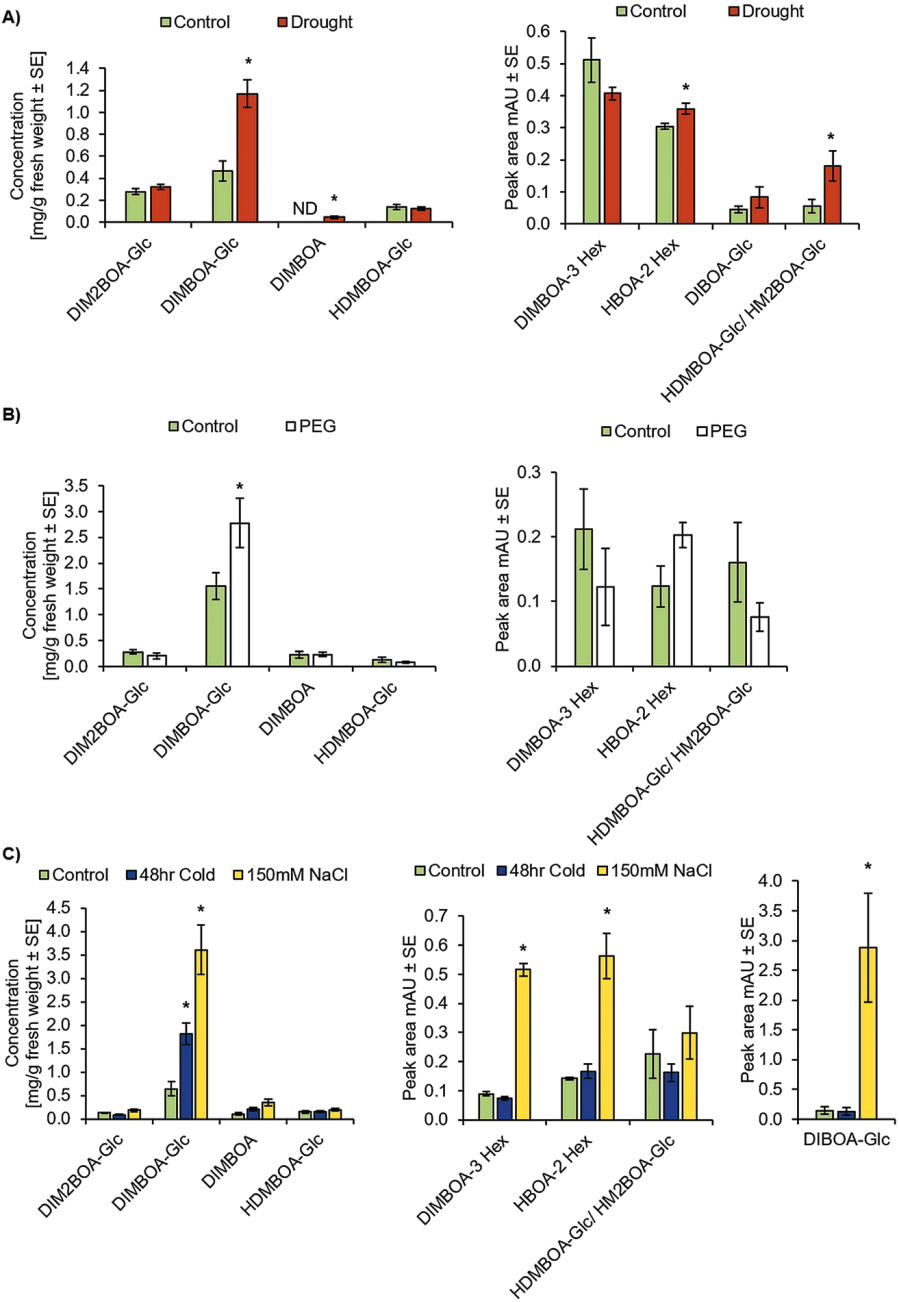
Environmental stresses induce BXD levels in wheat leaves. (A) BXD profile of plants grown under drought conditions. (B) BXD profile of plants affected by polyethylene glycol (PEG) treatment. (C) BXD levels of plants grown under either low temperature or salt stress conditions. Asterisks indicate a significant difference, *P*<0.05 by Student’s *t*-test (*n*=7–8). ND, not detected.

We examined whether several abiotic stresses affected the BXD levels, by exposing wheat seedlings to drought, salt, and low-temperature conditions, and measured the metabolites in the shoots. On the basis of current knowledge about *TaMYB31* (Bi *et al.*, 2016; Zhao *et al.*, 2018), we hypothesized that BXD levels should also be induced under drought conditions. As shown in Fig. 4A, drought treatment caused elevations in DIMBOA-Glc, DIMBOA, HBOA-2 Hex, and HDMBOA-Glc/HM2BOA-Glc levels in wheat plants. To exclude the low water potentials that reduce water availability, we performed a dehydration stress experiment using PEG-infused agar plates. Thus, the plants that were grown on PEG-infused agar plates accumulated a significantly higher level of DIMBOA-Glc (Fig. 4B). DIBOA-Glc was not detected in plants grown on plates for the PEG treatment. Interestingly, the control plants grown in soil for the drought treatment did not accumulate DIMBOA, while this compound was present in the control plants of the PEG treatment, which might be a result of the interaction of the plant with the soil microbiota. To test whether salt stress affects BXD biosynthesis, seeds were sown in a 150 mM sodium chloride solution. The results presented in Fig. 4C demonstrate that salinity greatly induced BXD levels, specifically those of DIMBOA-Glc, DIMBOA-3 Hex, HBOA-2 Hex, and DIBOA-Glc. Notably, BXD levels were highly abundant under high salt conditions, which might be an indication of their role in adaptation to this condition. Compared with salt stress, a low-temperature treatment of 4 °C showed only induction of one BXD, DIMBOA-Glc (Fig. 4C). The results suggested that BXD metabolites are modified in response to environmental stress conditions. To test whether the accumulation of BXDs under abiotic stress conditions corresponds to levels of *TaMYB31*, we performed a gene expression analysis of wheat grown under drought and salt stress conditions. Under drought, *TaMYB31* transcript levels were induced 2-fold (Supplementary Fig. S5A), while no changes were observed under salinity (Supplementary Fig. S5C). Moreover, we analyzed the expression of *Bx1* and *Bx4*, two genes that were activated by TaMYB31 protein (Fig. 3), and found a significant gene induction under drought conditions of 1.5- and 3-fold, respectively (Supplementary Fig. S5B). These findings emphasize the involvement of *TaMYB31* in the regulation of the BXD pathway under both biotic and abiotic stress conditions. However, the roles played by the different BXDs in abiotic stresses warrant further investigation.

### Enrichment analysis of *TaMYB31* and *Bx* genes with a MYB-binding site

We next selected wheat expression data from publicly available sources to examine the co-expression of the *TaMYB31* and BXD biosynthesis pathway genes. A soft thresholding power was determined for each dataset according to an analysis of scale-free topology for multiple soft thresholding powers (Zhang and Horvath, 2005). The *Bx* genes were divided into two groups: (i) *Bx* genes containing a *TaMYB31* promoter-binding site (*TaMYB31* target genes) and (ii) *Bx* genes that do not contain a *TaMYB31* promoter-binding site (*TaMYB31* non-target-genes). In total, 73 genes from the BXD pathway in wheat were used for prediction, 25 *Bx* genes in the *TaMYB31* target gene group, and 48 in the *TaMYB31* non-target gene group (Supplementary Table S8). Then, we identified enriched modules from six datasets associated with *Bx* genes using the WGCNA method. As presented in Table 2, the network analysis resulted from a higher number of clusters of *Bx* genes with *TaMYB31* target genes than non-target genes (five versus three clusters, respectively). Notably, the number of enriched clusters was higher in the *TaMYB31* target gene group, although it is represented by only 34.2% of the total *Bx* genes. Overall, four datasets showed enriched clusters of *TaMYB31* target genes, including pathogens (one cluster from powdery mildew and two clusters from *Fusarium pseudograminearum*) and abiotic stresses (one cluster of cold and one cluster of drought and heat stresses). These results strengthen our finding that *TaMYB31* is involved in BXD biosynthesis via the regulation of *Bx* gene expression.

**Table 2.** A network analysis of *Triticum aestivum* RNA-seq data using publicly available wheat expression sources

Dataset	Treatment	Clusters	Clusters *TaMYB31*/*Bx* genes	Enriched clusters (*TaMYB31* target genes)	Enriched clusters (*TaMYB31* non-target genes)
SRP041017	Powdery mildew	61	16	1	1
SRP043554	Cold	20	4	1	0
SRP045409	Drought and heat	49	17	1	0
SRP048912	*Fusaruim pseudograminearum*, 2 d	37	17	2	1
SRP068165	PEG	207	28	0	1
SRP078208	*Fusaruim pseudograminearum*, 3 d	402	27	0	0

*TaMYB31* target genes were predicted *in silico* (PlantRegMap).

## Discussion

BXDs have been a topic of growing interest for the last several decades due to their ecological potential in crop protection and pest management, as well as their abundance in cereal crops (Niculaes *et al.*, 2018; Zhou *et al.*, 2018). However, there are many open questions regarding BXD function under various stress conditions, the transcriptional regulation of genes, and transport between organelles and tissues. Moreover, most of the knowledge related to this pathway was generated from maize, while there are many open questions regarding other plant species. Here, we combined a transcriptomic dataset, gene silencing, and metabolic analysis to identify the potential TFs that may regulate this pathway. The expression of the gene encoding TaMYB31, a R2R3-MYB family TF, showed that it is up-regulated upon both aphid and caterpillar feeding (Table 1). MYBs are a large family of TFs, highly abundant in plants. They are known to be involved in controlling various cell processes, from responses to biotic and abiotic stresses to plant development, cell differentiation, and metabolism, as well as defense (Ambawat *et al.*, 2013). A large-scale *in silico* identification of the MYB family in wheat revealed 218 sequences out of 464 MYB contigs, and singlets were found to be potential MYB proteins, including 1R-MYB, R2R3-MYB, 3R-MYB, and 4R-MYB subfamilies (Cai *et al.*, 2012; Zhang *et al.*, 2012). Currently, full-length sequences of at least 73 *TaMYB* genes are known and reported to play a role in drought stress and phloem-based defense through the regulation of callose biosynthesis and ethylene signaling (Baloglu *et al.*, 2014; Zhai *et al.*, 2017), yet most of their functions have not been characterized. Our study showed that transient gene silencing of *TaMYB31* reduced the BXD levels in a heterologous plant system, which was correlated with induction of pest susceptibility relative to the control (Figs 1, 2), and exposed possible interactions between the TaMYB31 protein and *Bx* gene promoters in plants (Fig. 3). Altogether, we discovered that *TaMYB31* is a crucial regulator of BXD biosynthesis in wheat and might play a dual role under biotic and abiotic stresses.

The three subgenomes differentially contribute to BXD biosynthesis in hexaploid wheat, where *Bx* homologs in the B subgenome mainly contribute to the gene expression of this pathway (Nomura *et al.*, 2005; Powell *et al.*, 2017). This supports our transcriptomic analysis of the tetraploid wheat (*T. turgidum* ssp. *durum*) cv. Svevo, which found that *MYB31,* located on chromosome 5 of subgenome B, increased its expression upon aphid and caterpillar infestation (Table 1). Similarly, the expression analysis of *TaMYB31* genes in the leaves, roots, and flowering spikes of the hexaploid wheat Chinese Spring showed a higher transcript level of subgenome B than other homoeologous genes (Zhao *et al.*, 2018). We suggest that the TraesCS5B02G226100 gene in Chinese Spring hexaploid wheat and the TRITD5Bv1G134610 gene in durum wheat are the predominant *MYB31* isoforms.

BXD compounds play a role in plant defense against pests. They are highly abundant in young wheat leaves when the plants are most vulnerable to herbivore damage and decline upon plant development when other defense strategies have evolved (Cambier *et al.*, 2000; Kohler *et al.*, 2015; Batyrshina *et al.*, 2020; Singh *et al.*, 2021). The susceptibility of maize recombinant inbred lines to *R. maidis* aphids was negatively correlated with DIMBOA-Glc levels (Meihls *et al.*, 2013). The resistance of maize and wheat plants to aphids is affected by callose deposition, which is related to DIMBOA and DIMBOA-Glc abundance (Ahmad *et al.*, 2011; Betsiashvili *et al.*, 2015; Li *et al.*, 2018). Moreover, DIMBOA and its breakdown product MBOA were shown to increase the levels of detoxification enzymes in the European corn borer, *Ostrinia nubilalis* (Feng *et al.*, 1991, 1992), while DIMBOA in maize roots showed both feeding deterrence and toxicity effects on Western corn rootworm (*Diabrotica virgifera virgifera)* larvae (Xie *et al.*, 1990, 1992). Our results revealed that a reduction in *TaMYB31* gene expression (Figs 1A, 2A) resulted in a reduction in BXD accumulation (Fig. 1B) and increased insect performance (Fig. 2B). These results agree with previous reports indicating that BXD levels, especially of DIMBOA-Glc and HDMBOA-Glc, determined insect herbivory performance (Oikawa *et al.*, 2004; Ahmad *et al.*, 2011; Glauser *et al.*, 2011; Meihls *et al.*, 2013; Mijares *et al.*, 2013; Shavit *et al.*, 2018). BXDs negatively impacted the productivity of generalist spider mites that fed on maize (Bui *et al.*, 2018) and wheat plants (Shavit *et al.*, 2022). Moreover, in response to mite infestation of maize plants, to either *Oligonychus pratensis* or TSSM, six *Bx* genes were induced (Bui *et al.*, 2018), while some genes (*Bx10–Bx12)* shared quantitative trait locus intervals with TSSM resistance genes (Bui *et al.*, 2021).

Increases in BXD antifeedant, antixenotic, antimicrobial, and allelopathic properties under biotic stresses are frequently reported (Niemeyer, 2009; Li *et al.*, 2018), while the role they play under environmental changes is not. The *TaMYB31* gene was previously reported to be highly expressed in wheat under drought growth conditions, involving cuticle formation (Bi *et al.*, 2016). The ectopic expression of this gene in Arabidopsis plants revealed its involvement in drought resistance and wax biosynthesis regulation (Zhao *et al.*, 2018). However, the role of BXDs under drought conditions is unknown. Our experiments confirmed that the transcript levels of *TaMYB31* and *Bx* genes (*Bx1* and *Bx4*) are highly induced under drought (Supplementary Fig. S5A, B), which in turn may cause an elevation in BXD levels (Fig. 4A). Although TaMYB31 activates promoters of genes from the early metabolic steps of the BXD pathway, the BXD compositions were slightly different between the VIGS assay and the drought. Interestingly, *TaMYB31*-silenced plants significantly lost downstream BXD compounds (Fig. 1), while early pathway intermediates were affected by drought stress (Fig. 4A). Nevertheless, DIMBOA-Glc, the main BXD compound in wheat leaves (Zhang *et al.*, 2021a), was affected by both conditions. These differences might result from the developmental stage since the BXD pathway is dependent on plant age. Another possibile reason for the differences between the BXD profile of the VIGS assay and the drought is that additional TFs might be up-regulated under these conditions, which may target other *Bx* genes. However, this requires further investigation. We also used a PEG-supplemented medium to test the dehydration treatment effect, which was shown to be an up-regulation condition for the *TaMYB31* gene level (Zhao *et al.*, 2018). Our results revealed elevated levels of DIMBOA-Glc in the leaves (Fig. 4B), supporting the expression analysis conducted by Zhao *et al.*, 2018. Similarly, transcriptome analysis of wheat leaves grown under PEG treatments for 2 h and 12 h showed up-regulation of *TaMYB31* and *Bx* genes. Their expression is differentiated by wheat variety and exposure time to stress conditions (Supplementary Table S9). Based on the lack of knowledge on BXD changes under abiotic stress conditions, we additionally evaluated the influence of low temperature and salinity in wheat plant tissues (Fig. 4C). In both conditions, the DIMBOA-Glc levels were increased, while the salinity treatment also showed a significant accumulation of three other BXDs, suggesting that salinity has a strong induction effect on BXD biosynthesis. However, gene expression of *TaMYB31* was not affected by salt stress (Supplementary Fig. S5C), suggesting an involvement of other TFs in this condition. Interestingly, cold stress highly induced *TaMYB31* and *Bx* genes, including *Bx3*, *Bx4*, and *Bx8/9* (Supplementary Table S9). Further investigation is required to provide new insights into the transcriptional regulation of genes and molecular mechanisms that caused changes under these environmental conditions. TFs control the entire life cycle of plants, from germination to seed development and from response to environmental cues and leaf senescence to programmed cell death (Agarwal *et al.*, 2011; Kim *et al.*, 2018; Cubría-Radío and Nowack, 2019; Li *et al.*, 2019; Crawford *et al.*, 2020; Xu *et al.*, 2020). The biosynthesis of specialized metabolite pathways is also regulated by specific TFs (Yang *et al.*, 2012; Barco and Clay, 2020). As shown in Fig. 4C, salt stress resulted in the elevation of BXD levels that did not show the involvement of *TaMYB31*. This indicated the involvement of other TFs in the regulation of the BXD pathway under salt stress. TF families such as WRKY, MYB, NAC, and bZIP have been reported to be involved in crop plants’ responses to salt stress. For example, wheat transcription factor genes *TaWRKY93* (Qin *et al.*, 2015) and *TaWRKY10* (Wang *et al.*, 2013) confer tolerance to salt stress in Arabidopsis and transgenic tobacco, respectively. The TFs TaMYB73 (He *et al.*, 2012) and TaNAC47 (Zhang *et al.*, 2016) were induced by NaCl in wheat seedlings, while ectopic expression in Arabidopsis improved salinity tolerance. Similarly, *TabZIP14-B* was up-regulated by several stress treatments, including salinity, and Arabidopsis plants overexpressing *TabZIP14-B* showed enhanced tolerance to salt stress (Zhang *et al.*, 2017). Another TF from the bZIP family, TabZIP15, is involved in improving salt tolerance in transgenic wheat lines, increasing above- and below-ground tissue fresh weight, height, and length, as well as decreasing oxidative stress contents (Bi *et al.*, 2021). Thus, the regulation of BXD biosynthesis under salt stress requires further investigation.

Biological processes in plant cells are regulated by sequence-specific TFs that function by binding transcriptional regulatory regions, such as promoters, enhancers, and terminators. Studies that have investigated this interaction exploit bioinformatic tools (Frith *et al.*, 2001, 2003; Bailey and Noble, 2003; Beckstette *et al.*, 2006; Turatsinze *et al.*, 2008; Grant *et al.*, 2011) for *de novo* motif discovery. The domain structure and activation properties of TaMYB31 were previously investigated (Bi *et al.*, 2016). Here, we used the Plant Transcription Factor database (Jin *et al.*, 2015, 2017; Tian *et al.*, 2020) to identify TaMYB31-binding sites in the 1 kb upstream of the start codon region of *Bx* genes. Then, we performed a network analysis that showed the co-expression of predicted target *Bx* genes with *TaMYB31* under environmental stress conditions such as cold, drought, and heat (Table 2), as well as biotic stress inducers such as fungal pathogens (Table 2). Furthermore, a multivariant analysis of the *MYB31*, *Bx*, and *IGPS* gene expression levels, generated from the transcriptomic dataset of Svevo seedlings subjected to 6 h of either *R. padi* aphid or *S. littoralis* caterpillar feeding, supported these findings (Shavit *et al.*, 2022). The heatmap presented in Supplementary Fig. S6 showed that both *MYB31* homologs are co-expressed, with 36 genes associated with this pathway.

Several methods are commonly used for the functional analysis of protein–DNA binding sites, such as yeast-one hybrid or dual-luciferase assays (Li and Herskowitz, 1993; Wang and Reed, 1993; Hellens *et al.*, 2005; Reece-Hoyes and Walhout, 2012). Here, we used a dual fluorescence analysis for the *in planta* evaluation of *Bx* promoter activation by TaMYB31. We tested six predicted/tested *Bx* promoters and found that *TaBx1* and *TaBx4* showed significant activation by TaMYB31. Furthermore, we selected a DNA fragment of a *TaKCS1* cuticle-related gene promoter with three possible binding sites for TaMYB31 (Supplementary Table S10). As shown in Fig. 3, the predicted part of the *TaKCS1* promoter confirmed regulation by TaMYB31, which emphasizes the accuracy of the dual fluorescence system. Previous studies have shown that TF target genes are not always sequential in the biosynthetic steps. For example, co-expression-based gene regulatory networks suggested four TFs that may regulate *Bx* genes, where each TF was linked to several *Bx* genes (not in a sequential manner) and varied between networks (Zhou *et al.*, 2020). The TF genes *ZmbLHL57* and *ZmWRKY34* are suggested to regulate *Bx* genes in systemic leaves under *M. separata* caterpillar feeding, where *ZmbLHL57* was co-expressed with *Bx2* and *ZmWRKY34* with *Bx6* and *Bx10–11*, respectively (Malook *et al.*, 2019). Overall, our findings indicated that at least two metabolic steps of the BXD pathway, catalyzed by TaBx1 and TaBx4, are activated by the TaMYB31.

### Conclusions

This is the first report to identify a TF regulating BXD biosynthesis in wheat and to characterize its role *in planta*. In addition, we observed that BXDs are involved in numerous biotic and abiotic stresses, while their role under salinity and cold stress requires further investigation. The next step for better understanding BXD functions and *TaMYB31* regulation is to generate stable knockout mutants and test their BXDs and resistances under various and combined stress conditions and growth stages.

## Supplementary data

The following supplementary data are available at *JXB* online.

Table S1. The oligonucleotide sequences that were used in this study.

Table S2. The *in silico* prediction results of *Bx* genes for the presence of TaMYB31-binding sites.

Table S3. Wheat expression database information used for network analysis.

Table S4. Scale-free topology and WGCNA results of selected datasets.

Table S5. An overview of TF family functions in plants.

Table S6. Candidate TF IDs.

Table S7. TF-binding site positions on *Bx* promoters.

Table S8. List of selected *Bx* genes and whether they contain TaMYB31-binding sites on the promoters.

Table S9. Expression of *TaMYB31* and *Bx* genes under abiotic stress conditions.

Table S10. Predicted binding sites of the *TaKCS1* promoter for TaMYB31 regulation.

Fig. S1. Scheme of the benzoxazinoid (BXD) biosynthetic pathway.

Fig. S2. The *TaMYB31* coding region sequence was optimized to eliminate the cleavage sites of inner IIS restriction (*Bsa*I and *Bsm*BI).

Fig. S3. Photobleaching phenotype of *BSMV::PDS*.

Fig. S4. Herbivore-induced BXD levels of *TaMYB31*-silenced plants relative to the *BSMV::empty* vector plants.

Fig. S5. Gene expression levels of *TaMYB31* and *Bx* genes under abiotic stress conditions.

Fig. S6. Heatmap of the multivariant analysis of the transcriptomic data of *TtBx* and *TtMYB31*.

erac204_suppl_supplementary_figures_S1-S6

erac204_suppl_supplementary_tables_S1-S10

## Data Availability

All datasets generated for this study are included in the article and its supplementary data published online
