# Supplementary material for: The transcription factor TaMYB31 regulates the benzoxazinoid biosynthetic pathway in wheat
Source: J Exp Bot. 2022 May 12;73(16):5634–49. doi: 10.1093/jxb/erac204 (PMC9467655; doi:10.1093/jxb/erac204)
Supplement: erac204_suppl_supplementary_figures_S1-S6 [file erac204_suppl_supplementary_figures_s1-s6.pdf]

Supplementary Figure S1.

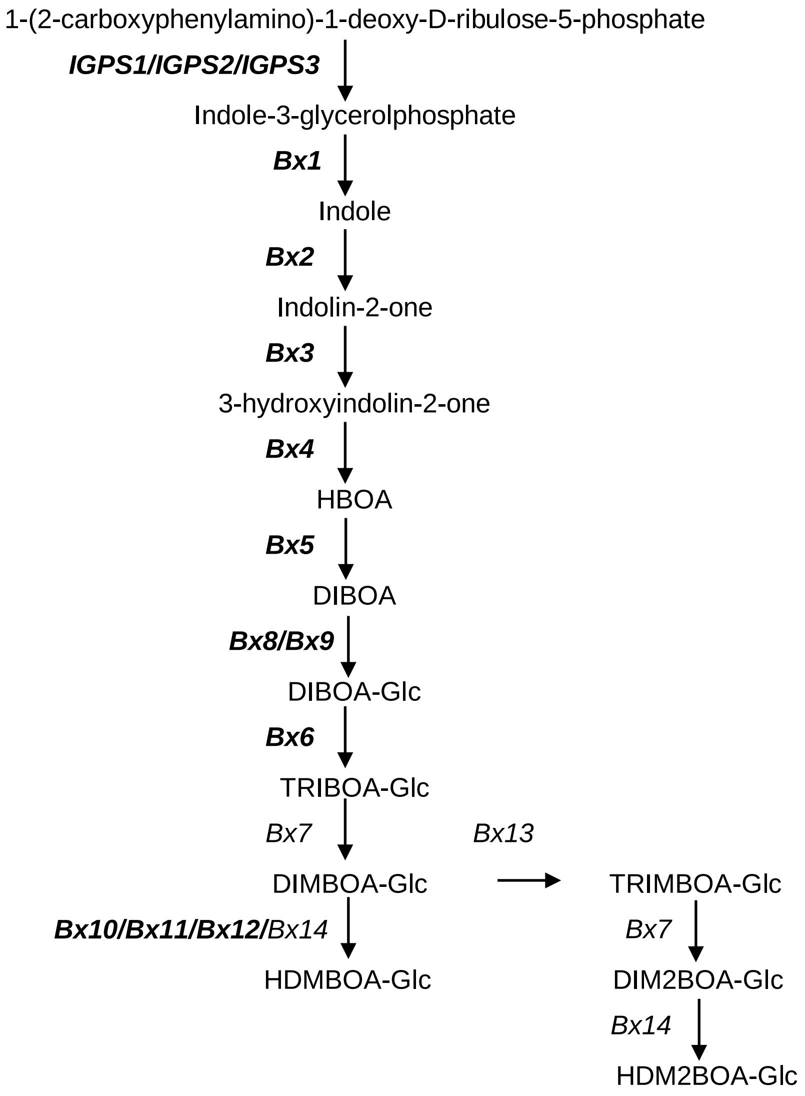

Supplementary Figure S1. Scheme of the benzoxazinoid (BXD) biosynthetic pathway. In bold fonts are annotated genes in wheat.

Supplementary Figure S2.

A)

```
TaMYB31_CDS_original     ATGGGGAGGCCTCCGTGCTGCGACAAGGTGGGCGTCAAGAAGGGGCCGTGGACGCCGGAG 60
TaMYB31_CDS_optimized    ATGGGGAGGCCTCCGTGCTGCGACAAGGTGGGCGTCAAGAAGGGGCCGTGGACGCCGGAG 60
                         ************************************************************

TaMYB31_CDS_original     GAGGACCTCATGCTCGTCTCCTACATCCAGGAGCACGGCCCAGGGAACTGGCGCGCCGTG 120
TaMYB31_CDS_optimized    GAGGACCTCATGCTCGTTTCCTACATCCAGGAGCACGGCCCAGGGAACTGGCGCGCCGTG 120
                         ***************** ******************************************

TaMYB31_CDS_original     CCGACCAACACCGGGCTGATGCGGTGCAGCAAGAGCTGCCGGCTTCGGTGGACCAACTAC 180
TaMYB31_CDS_optimized    CCGACCAACACCGGGCTGATGCGGTGCAGCAAGAGCTGCCGGCTTCGGTGGACCAACTAC 180
                         ************************************************************

TaMYB31_CDS_original     CTTCGGCCGGGGATCAAGCGCGGCAACTTCAACGACCAGGAGGAGAAGCTCATCGTCCAC 240
TaMYB31_CDS_optimized    CTTCGGCCGGGGATCAAGCGCGGCAACTTCAACGACCAGGAGGAGAAGCTCATCGTCCAC 240
                         ************************************************************

TaMYB31_CDS_original     CTCCAGGCGCTGCTCGGCAACCGTTGGGCGGCGATAGCGTCCTACTTGCCCGAGAGGACG 300
TaMYB31_CDS_optimized    CTCCAGGCGCTGCTCGGCAACCGTTGGGCGGCGATAGCGTCCTACTTGCCCGAGAGGACG 300
                         ************************************************************

TaMYB31_CDS_original     GACAACGACATCAAGAACTACTGGAACACCCATCTCAAGAAGAAGCTCAAGAAGATGCAG 360
TaMYB31_CDS_optimized    GACAACGACATCAAGAACTACTGGAACACCCATCTCAAGAAGAAGCTCAAGAAGATGCAG 360
                         ************************************************************

TaMYB31_CDS_original     GACGCCGGAGGGAACGACGGCGGCTCGGAGGGCGCCGGCGCCGCCGGTGTCGGTGGTGGC 420
TaMYB31_CDS_optimized    GACGCCGGAGGGAACGACGGCGGCTCGGAGGGCGCCGGCGCCGCCGGTGTCGGTGGTGGC 420
                         ************************************************************

TaMYB31_CDS_original     GTCGCCAAGGCGGCCGCTCCCAAAGGGCAGTGGGAGCGCCGGCTGCAGACGGACATCCAC 480
TaMYB31_CDS_optimized    GTCGCCAAGGCGGCCGCTCCCAAAGGGCAGTGGGAGCGCCGGCTGCAGACGGACATCCAC 480
                         ************************************************************

TaMYB31_CDS_original     ACTGCGCGGCAGGCCCTGCGCGACGCGCTCTCTCTAGAGCCCTCACAGCCCGCCGCGCTG 540
TaMYB31_CDS_optimized    ACTGCGCGGCAGGCCCTGCGCGACGCGCTCTCTCTAGAGCCCTCACAGCCCGCCGCGCTG 540
                         ************************************************************

TaMYB31_CDS_original     GCGGCGCCGGCGCTGCCGACGCCTCCGGGGTCGGTGACGACGTACGCGTCGAGCGCGGAC 600
TaMYB31_CDS_optimized    GCGGCGCCGGCGCTGCCGACGCCTCCGGGGTCGGTGACGACGTACGCGTCGAGCGCGGAC 600
                         ************************************************************

TaMYB31_CDS_original     AACATCGCGCGCCTACTGGAGGGCTGGATGCGCCCCGGGAGCAGCAGCAAGGGGCCGGAG 660
TaMYB31_CDS_optimized    AACATCGCGCGCCTACTGGAGGGCTGGATGCGCCCCGGGAGCAGCAGCAAGGGGCCGGAG 660
                         ************************************************************

TaMYB31_CDS_original     GCGTCGGGGTCGACGTCGTCCACTACGGCGACGACCCGCCAGCAGCCACAGTGCTCCTGG 720
TaMYB31_CDS_optimized    GCGTCGGGGTCGACGTCGTCCACTACGGCGACGACCCGCCAGCAGCCACAGTGCTCCTGG 720
                         ************************************************************

TaMYB31_CDS_original     GATGGCGCGGCGTCCGCGTCGGCGAGTCACAGCGGCGGCGCGGCCGGGGCGGCAGCAGCG 780
TaMYB31_CDS_optimized    GATGGCGCGGCGTCCGCGTCGGCGAGTCACAGCGGCGGCGCGGCCGGGGCGGCAGCAGCG 780
                         ************************************************************

TaMYB31_CDS_original     CAGACTCCCGAGGGCTCGACCGAGACGAGCAAGCTGGCCGGCGCCGGGGCCGGCGGCGCC 840
TaMYB31_CDS_optimized    CAGACTCCCGAGGGCTCGACCGAGACTAGCAAGCTGGCCGGCGCCGGGGCCGGCGGCGCC 840
                         ************************** *********************************

TaMYB31_CDS_original     CCGCCGGCGTTCTCCATGCTGGAGAACTGGCTGCTTGACGACGGCATGGGGCACGGCGAG 900
TaMYB31_CDS_optimized    CCGCCGGCGTTCTCCATGCTGGAGAACTGGCTGCTTGACGACGGCATGGGGCACGGCGAG 900
                         ************************************************************

TaMYB31_CDS_original     GCGGGACTCATGGACGACGTGGTGCCACTAGGGGACCCCAGTGAGTTCTTCTAA       954
TaMYB31_CDS_optimized    GCGGGACTCATGGACGACGTGGTGCCACTAGGGGACCCCAGTGAGTTCTTCTAA       954
                         ******************************************************
```

B)

```
TaMYB31_CDS_original     MGRPPCCDKVGVKKGPWTPEEDLMLVSYIQEHGPGNWRAVPTNTGLMRCSKSCRLRWTNY 60
TaMYB31_CDS_optimized    MGRPPCCDKVGVKKGPWTPEEDLMLVSYIQEHGPGNWRAVPTNTGLMRCSKSCRLRWTNY 60
                         ************************************************************

TaMYB31_CDS_original     LRPGIKRGNFNDQEEKLIVHLQALLGNRWAAIASYLPERTDNDIKNYWNTHLKKKLKKMQ 120
TaMYB31_CDS_optimized    LRPGIKRGNFNDQEEKLIVHLQALLGNRWAAIASYLPERTDNDIKNYWNTHLKKKLKKMQ 120
                         ************************************************************

TaMYB31_CDS_original     DAGGNDGGSEGAGAAGVGGGVAKAAAPKGQWERRLQTDIHTARQALRDALSLEPSQPAAL 180
TaMYB31_CDS_optimized    DAGGNDGGSEGAGAAGVGGGVAKAAAPKGQWERRLQTDIHTARQALRDALSLEPSQPAAL 180
                         ************************************************************

TaMYB31_CDS_original     AAPALPTPPGSVTTYASSADNIARLLEGWMRPGSSSKGPEASGSTSSTTATTRQQPQCSW 240
TaMYB31_CDS_optimized    AAPALPTPPGSVTTYASSADNIARLLEGWMRPGSSSKGPEASGSTSSTTATTRQQPQCSW 240
                         ************************************************************

TaMYB31_CDS_original     DGAASASASHSGGAAGAAAAQTPEGSTETSKLAGAGAGGAPPAFSMLENWLLDDGMGHGE 300
TaMYB31_CDS_optimized    DGAASASASHSGGAAGAAAAQTPEGSTETSKLAGAGAGGAPPAFSMLENWLLDDGMGHGE 300
                         ************************************************************

TaMYB31_CDS_original     AGLMDDVVPLGDPSEFF    317
TaMYB31_CDS_optimized    AGLMDDVVPLGDPSEFF    317
                         *****************
```

Supplementary Figure S2. The *TaMYB31* coding region sequence was optimized to eliminate the cleavage sites of inner IIS restriction (*BsaI* and *BsmBI*). A) Alignment of coding region sequences; changed nucleotides are highlighted in red. B) Alignment of amino acid sequences.

Supplementary Figure S3.

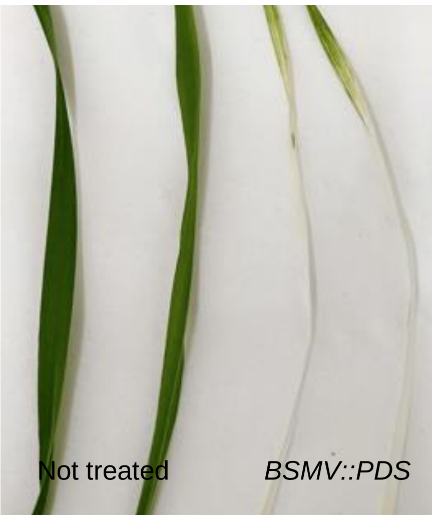

Photobleaching phenotype of *BSMV::PDS*.

Supplementary Figure S3. Photobleaching phenotype of *BSMV::PDS*.

Supplementary Figure S4.

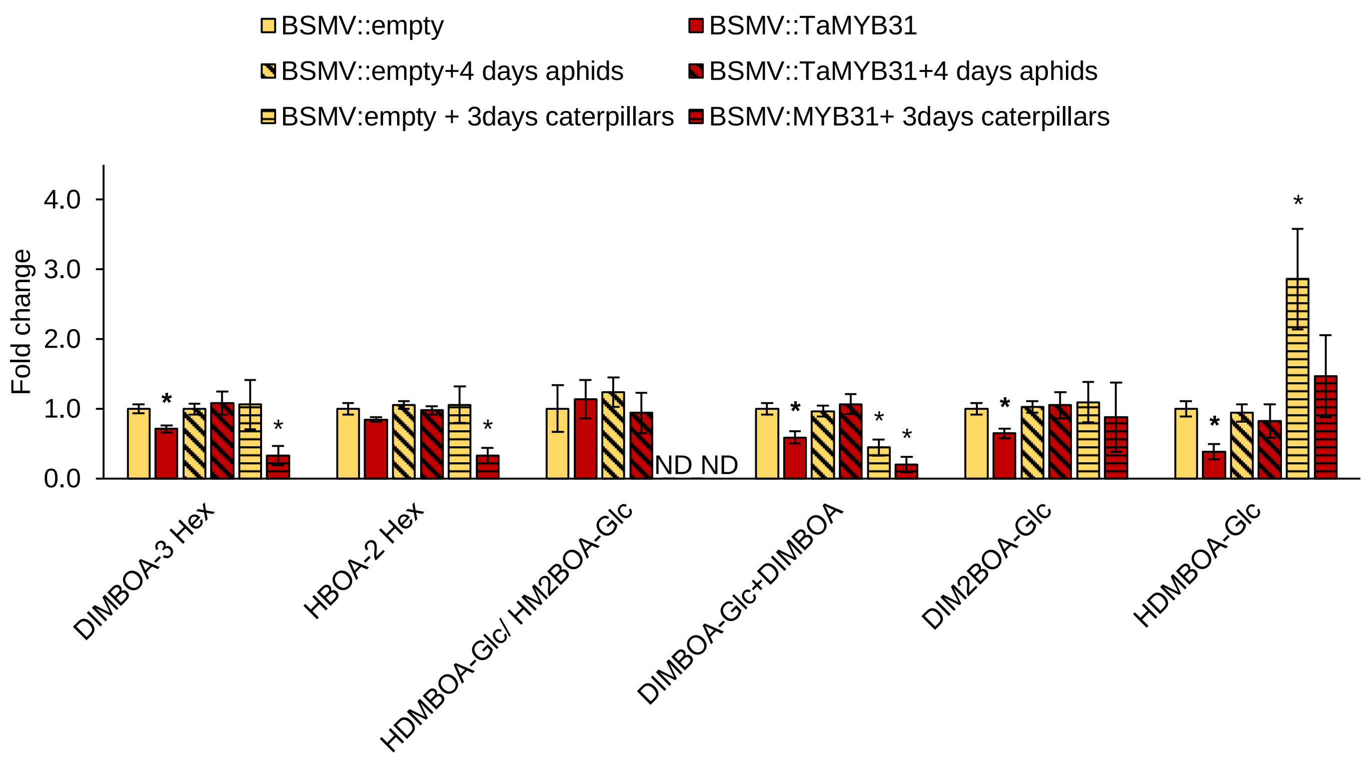

Supplementary Figure S4. Herbivore-inducible BXD levels of TaMYB31-silenced plants relative to the BSMV::empty vector plants. An asterisk indicates the values that were determined by Student's t-test relative to BSMV::empty vector plants $p < 0.05$.

Supplementary Figure S5.

A)

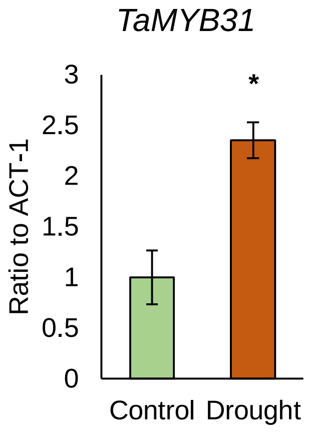

B)

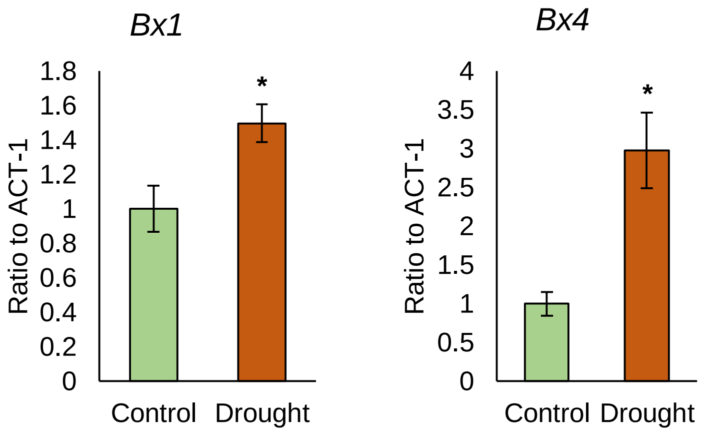

C)

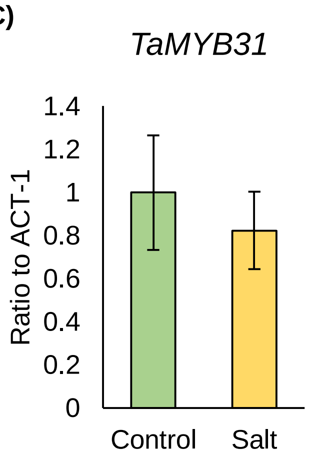

Supplementary Figure S5. Gene expression level of TaMYB31 and Bx genes under abiotic stress conditions. A) Transcript quantity of TaMYB31 under drought stress. B) Transcript quantity of selected Bx genes under drought stress. C) Transcript quantity of TaMYB31 under salt stress; An asterisk indicates the values that were determined by Student's t-test relative to control conditions, $p < 0.05$ (n=5).

Supplementary Figure S6.

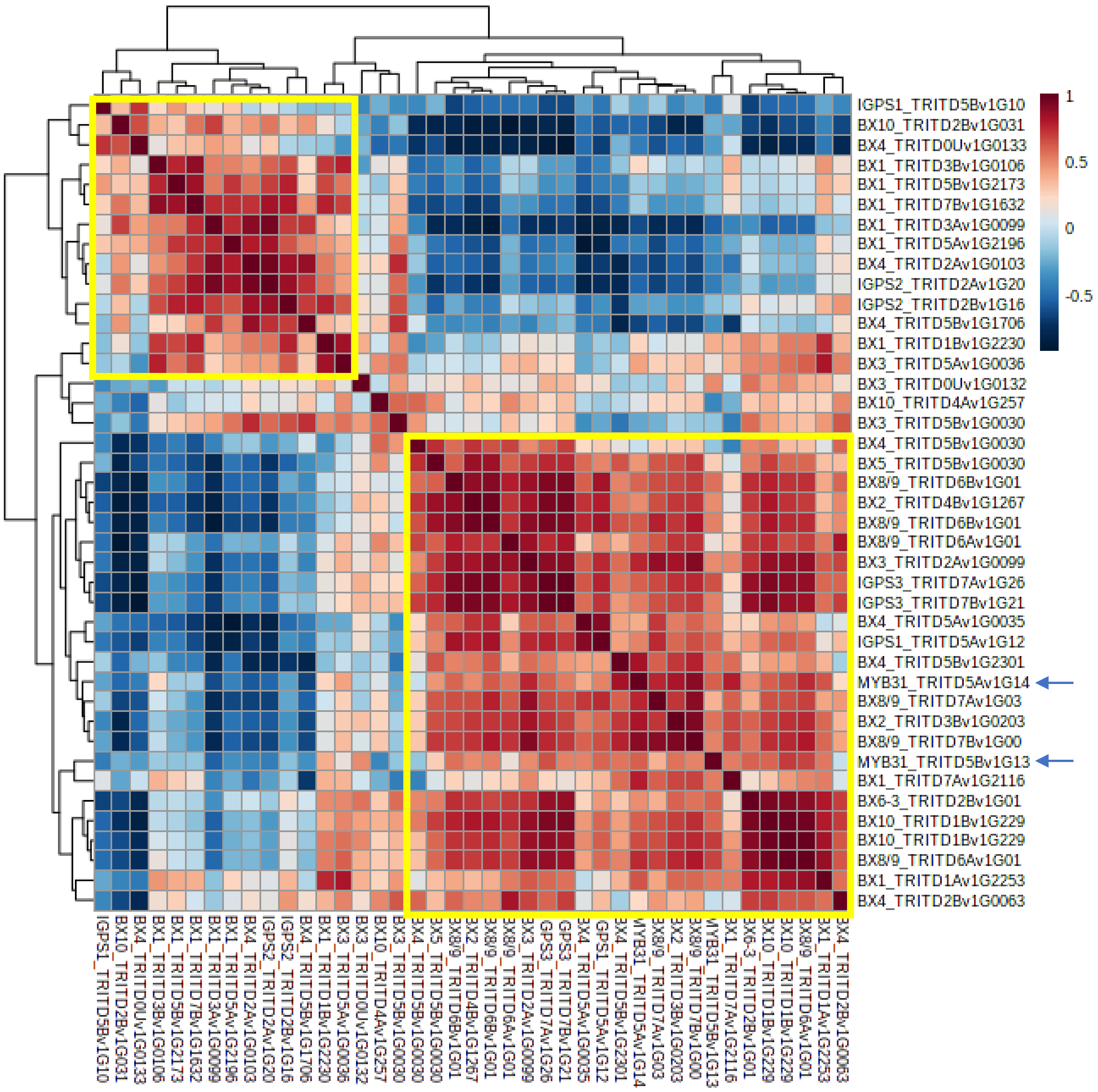

Supplementary Figure S6. Heatmap of the multivariant analysis of the transcriptomic data of *TtBx* and *TtMYB31* transcripts (clusters marked in yellow). Data were generated from Svevo leaves subjected to either *R. padi* or *S. littoralis* feeding for 6 h (Shavit *et al.*, 2022). Positive correlation coefficient values are in red, and negative correlation coefficient values are in blue. Blue arrows indicate *TtMYB31* genes.
